# Feasibility of Achieving Dose Constraints for Dysphagia Aspiration-Related Structures and Its Clinical Significance in Intensity-Modulated Radiotherapy Planning of Head and Neck Cancer

**DOI:** 10.7759/cureus.53769

**Published:** 2024-02-07

**Authors:** Karishma Agarwal, Piyush Kumar, Navitha S, Pavan Kumar, Ayush Garg, Jitendra Nigam, Silambarasan N.S

**Affiliations:** 1 Radiation Oncology, SRMS (Shri Ram Murti Smarak) Institute of Medical Sciences, Bareilly, IND

**Keywords:** dose constraints, dysphagia, dysphagia aspiration related structures, intensity modulated radiotherapy, head and neck cancers

## Abstract

Introduction

Dysphagia is commonly seen in patients with head and neck cancers after undergoing chemoradiotherapy and is often under-reported and also not given clinical importance. The quality of life of the patients can be significantly improved if the required dose constraints to the dysphagia aspiration-related structures (DARS) are achieved. The present study was conducted in order to determine the feasibility of achieving the dose constraints to DARS between the standard intensity-modulated radiotherapy (st-IMRT) arm and the dysphagia-optimized IMRT (do-IMRT) arm.

Material and methods

Sixty patients with head and neck cancer were recruited and randomized into two groups: In one group called the st-IMRT, constraints were not given to DARS, and in the other group called the do-IMRT, constraints were given to DARS. Treatment was given in the form of chemoradiation with a dose of 70 Gy in 35 fractions by IMRT technique, over seven weeks, 2 Gy per fraction along with weekly concurrent Cisplatin (35 mg/m^2^) in both the groups. Step and shoot IMRT setup was used for planning, and the system used for planning was Eclipse 13.6 (Varian Medical System, Inc., Palo Alto, CA, US); progressive resolution optimizer algorithm was used for optimization, and Anisotropic Analytical Algorithm algorithm was used for dose calculation. Truebeam was used for treatment delivery. DARS dosimetric parameters assessed were Dmean, V30, V50, V60, V70, D50, and D80. Radiation-induced toxicities to the skin, mucosa, larynx, salivary gland, and dysphagia and hematological toxicities were assessed in between both the groups during and after radiotherapy up to six months based on Common Terminology Criteria for Adverse Effects v5.0. p-values were calculated using the unpaired T-test.

Results

In the cohort of 60 patients with head and neck cancers, 95% were males. Dosimetric parameters of the planning target volume (PTV) were compared but were not found to be significant. In the dosimetry of the organs at risk, a p-value of some structures was found to be significant although the doses received were well within the tolerable limits in both arms. DARS dosimetry V60 and V70 of the inferior constrictor muscle was found to be statistically significant (p=0.01 and 0.008, respectively). V60 and V70 of larynx were also statistically significant (p=0.009 and 0.000, respectively). V70 and D50 of cricopharyngeus were found to be statistically significant (p=0.01 and 0.03, respectively), V30 and V60 for combined pharyngeal constrictor muscles were found to be statistically significant (p=0.02 and 0.01), and lastly, V60 for combined DARS was also significant (p=0.004). Post-treatment 33.3% of patients in the st-IMRT arm required Ryle’s tube placement. No grade 4 toxicities were seen in either arm regarding hematological toxicities, acute or chronic radiation-induced toxicities. In site-wise comparison of doses, the p-value was not found to be significant in patients with oropharyngeal and oral cavity carcinomas but was found to be statistically significant in the larynx and hypopharynx subsites.

Conclusion

The feasibility of achieving dose constraints to the DARS was seen in cases of laryngeal and hypopharyngeal cancers where the constrictor muscles were at a distance from the PTV. Further, the feasibility of achieving dose constraints may be seen in lower-dose prescriptions either in postoperative cases or in low-risk clinical target volume nodal volumes.

## Introduction

Chemoradiotherapy is the standard modality of treatment in most of the locally advanced head and neck cancers. The option of surgery may be difficult in view of severely mutilating surgeries and to obtain a good functional outcome. During chemoradiation, the commonest toxicity seen in head and neck cancer patients is mucositis. This affects oral food intake, weight loss, and quality of life. A previous study done in our institute by Khattar et al. [1] inferred that the delineation of oral mucosa as a pseudo-organ at risk (OAR) should routinely be done, which could help in the reduction of the severity of developing oral mucositis.

Chronic toxicities of chemoradiation in head and neck cancer patients commonly include xerostomia, dysphagia, laryngeal edema, and hypothyroidism. Chronic toxicities can be decreased by limiting the dose to the parotid glands, dysphagia aspiration-related structures (DARS), and larynx, respectively. Dysphagia has been reported as high as 44% by Van der Veen and Nuyts [2] where they describe various toxicities that occur in head and neck cancer patients and if these toxicities can be prevented by using intensity-modulated radiotherapy (IMRT) technique.

Recent studies have shown that aspiration in association with dysphagia is common after chemoradiotherapy which is under-reported and has not been given clinical importance [3,4]. The quality of life of the patients can be significantly improved if the required dose constraints to the DARS are achieved. A study was done earlier in our institute to evaluate doses to DARS where patients were divided into two groups, one group of patients were planned by the three-dimensional conformal radiotherapy (3D-CRT) technique and the other group by intensity-modulated radiotherapy (IMRT) technique. The study showed that IMRT can help reduce the doses received by the DARS as compared to 3D-CRT [5]. The present study is the extrapolation of the previous thought process to decrease the morbidity of dysphagia by IMRT technique. Through this study, we endeavor to check the feasibility of achieving the dose constraints to DARS which are involved in the process of swallowing.

## Materials and methods

The study was conducted after approval from the Institutional ethical committee, protocol no. SRMSIMS/IEC/2020-21/033. In this study, 60 patients with head and neck cancers were recruited who had not undergone any prior treatment of head and neck cancers. The inclusion criteria were age above 18 years with a Karnofsky Performance Scale (KPS) of more than 70 with normal blood parameters, normal echocardiogram with squamous cell carcinoma as the histopathology. Chemoradiation was planned with a dose of 70 Gy in 35 fractions by intensity-modulated radiotherapy (IMRT) technique, over seven weeks, 2 Gy per fraction along with weekly concurrent Cisplatin (35 mg/m^2^) in both groups. The patients were then randomized by simple randomization into two groups: in the first group, standard IMRT (st-IMRT) where constraints were not given to the DARS; and in the second group, dysphagia-optimized arm (do-IMRT) where constraints were given to DARS.

Radiotherapy planning and technique

Immobilization and Simulation

The patients were made to lie down on a base plate in an extended neck position and immobilized using a five-point thermoplastic cast, along with a neck rest. Contrast-enhanced CT (CECT) scan and radiotherapy planning (RTP) scan of 3 mm slice thickness were obtained with radio-opaque fiducial markers.

Delineation of Structures

Gross tumor volume (GTV): Gross disease including the primary tumor and enlarged lymph nodes as seen on clinical examinations and imaging modalities.

Clinical target volume (CTV) primary: Included whole of the primary subsite of GTV.

CTV nodal: Defined as the draining nodal region related to the primary. Department protocol follows guidelines of the delineation of neck node level for head and neck tumors as described by Gregoire et al. [6].

CTV final: Included CTV primary and CTV nodal.

Planning target volume (PTV): A symmetrical margin of 5 mm was taken from CTV to account for patient setup error (as per institutional protocol).

Delineation of Organs at Risk (OARs) Structures

The left and right parotid glands, spinal cord, brain stem, right and left eyes, right and left lens, optic chiasma, right and left optic nerve, right and left cochlea, lips, and mandible were delineated as per DAHANCA [7]. guidelines. An isotropic expansion of 5 mm was given for the planning risk volume (PRV) spine and 3 mm for the PRV brainstem.

​​Dysphagia Aspiration-Related Structures (DARS)

The structures of swallowing or “dysphagia aspiration-related structures” were identified as suggested by Christianen et al. [8]. The DARS included the following: constrictor muscles (superior (SCM), middle (MCM), inferior (ICM)), base of tongue (BOT), larynx, and cricopharyngeus muscle/upper esophageal sphincter (UES). Each DARS was delineated, and a combined volume for the constrictor muscles (CM) was generated; similarly, a combined volume for all the swallowing structures was also generated (DARS).

Dose Constraints to OARs and DARS

The dose constraints to the OARs were based on Radiation Therapy Oncology Group (RTOG) and Quantitative Analyses of Normal Tissue Effects in the Clinic (QUANTEC) criteria with the Dmax<54 Gy to PRV brainstem, Dmax<50 Gy to the PRV spine, Dmax<55 Gy to the optic chiasma, Dmax<70 Gy or 1cc<75 to the mandible, Dmax<55 Gy to the optic nerves, Dmax<50 Gy (0.03cc) to the right and left eyes, Dmax<7 Gy (0.03cc) to the right and left lens, Dmean<26 Gy to the parotids, Dmean<45 Gy to the right and left cochlea, and Dmean<30 Gy to the lips. In the st-IMRT arm, constraints were not given to the DARS, while in the do-IMRT arm, constraints were given to the DARS. The constraints given to the DARS were Dmean of 63 Gy to the SCM, MCM, BOT, and combined DARS, Dmean of 56 Gy to the ICM, 50 Gy to the combined constrictor muscles, 55 Gy to the larynx, and 50 Gy to the UES.

Radiotherapy Planning

Coplanar 7-9 fields around the isocenter using isotropic gantry angles were used and were adjusted slightly to avoid the beam entry through OARs. In the next step of fluence optimization, the dose coverage minimum and maximum required for PTV and dose tolerance to OARs were defined as per QUANTEC and RTOG.

Plan Evaluation

PTV dosimetric parameters were evaluated as follows: V95%, D95, Dmax, Dmean, conformity index (CI), and homogeneity index (HI). The mean dose and maximum dose for individual structures of swallowing, and also for combined swallowing structures, were tabulated for st-IMRT and do-IMRT groups.

Delivery of Image-Guided Radiotherapy

Portal imaging was matched with that of planning a digitally reconstructed radiograph (DRR) for aligning the bony structure. The shift in lateral, longitudinal, and vertical axes on matching was corrected. After that, a cone-beam CT scan (CBCT) was taken for soft tissue and planning target volume matching. Daily KV imaging and weekly CBCT were taken for correction of setup errors.

Assessment of toxicity

Radiation toxicity (skin, mucosal, laryngeal, salivary gland, and hematologic toxicity) was assessed by Radiation Therapy Oncology Group (RTOG) acute and late morbidity scoring criteria.

Patients were assessed weekly during chemoradiation for assessment of acute radiation reactions as per acute RTOG morbidity criteria from the day of starting radiotherapy and up to 90 days.

RTOG late morbidity criteria were used to assess late radiation-induced reactions applicable from 90 days onward.

During treatment, weekly and thereafter monthly assessments were done using Common Terminology Criteria for Adverse Effects (CTCAE) (v5.0).

For dysphagia, the patients were assessed weekly during treatment, at the end of the treatment, and thereafter monthly for up to six months. CTCAE (v5.0) subjective grading was used for assessment:

Grade I: Symptomatic, able to eat a regular diet.

Grade II: Symptomatic and altered eating/swallowing.

Grade III: Severely altered eating/swallowing, tube feeding, and total parenteral nutrition.

Grade IV: Life-threatening consequence, urgent intervention indicated.

Statistical analysis

Collected data was analyzed using standard statistical methods and software to calculate the level of significance using the “p” value with the unpaired T-test.

## Results

In the entire cohort of 60 patients, 57 (95%) patients were male. The male-to-female ratio was 19:1, while the age ranged between 18 and 69 years. In the st-IMRT arm, the mean age was 51 years and the median age was 54 years. In the do-IMRT arm, the mean age was 54.1 years and the median age was 55 years. Table 1 shows the clinical symptoms at the time of presentation, out of which dysphagia, ulcer, and pain were the most common symptoms in both arms.

**Table 1:** Clinical symptoms at presentation IMRT: intensity-modulated radiotherapy, do-IMRT: dysphagia-optimized IMRT, st-IMRT: standard IMRT.

Symptoms	st-IMRT n (%)	do-IMRT n (%)
Swelling	8 (26.6%)	13 (43.3%)
Ulcer	14 (46.6%)	10 (33.3%)
Pain	11 (36.6%)	13 (43.3%)
Hoarseness	5 (16.6%)	11 (36.6%)
Difficulty breathing	0 (0%)	1 (3.3%)
Dysphagia	12 (40%)	14 (46.6%)
Difficulty in protruding tongue	2 (6.6%)	1 (3.3%)

Table 2 shows the primary tumor site distribution in both arms, out of which the most number of cases was of oropharyngeal carcinoma.

**Table 2:** Primary tumor site-wise distribution IMRT: intensity-modulated radiotherapy, do-IMRT: dysphagia-optimized IMRT, st-IMRT: standard IMRT.

Site	st-IMRT n (%)	do-IMRT n (%)
Oropharynx	17 (56.6%)	10 (33.3%)
Oral cavity	8 (26.6%)	7 (23.3%)
Larynx	3 (10.2%)	9 (30%)
Hypopharynx	2 (6.6%)	4 (13.3%)

All the patients were of squamous cell carcinoma, out of which the most common grade of differentiation was moderate. Seventeen (28.3%) patients had well-differentiated histopathology, while 40 (66.6%) patients had moderately differentiated histopathology and three (5%) patients had poorly differentiated histopathology. In the entire cohort of patients, 46 (76.6%) were of stage IV and 10 (16.6%) were of stage III, while only four (6.6%) were of stage II.

Treatment compliance

In the st-IMRT arm, nine (30%) patients received five to seven cycles of chemotherapy, while in the do-IMRT arm, 21 (70%) patients received five to seven cycles of chemotherapy. However, no statistically significant difference was found in both arms.

Hematological toxicities

No toxicities were seen in 19 (63.3%) patients in the st-IMRT arm and 25 (83.3%) in the do-IMRT arm. No grade 4 toxicities were seen in any of the hematological parameters in either of the arms.

Ryle’s tube dependence

During treatment, lesser patients needed Ryle’s tube in the do-IMRT arm, three (10%), than in the st-IMRT arm patients, seven (23.3%). Post-treatment, 10 (33.3%) patients in the st-IMRT arm needed Ryle’s tube, while in the do-IMRT arm, no Ryle’s tube dependence was seen.

In the st-IMRT arm, 20 (66.6%) patients showed a complete response, eight (26.6%) patients showed a partial response, and two (6.6%) patients had a progressive disease, while in the do-IMRT arm, 26 (86.6%) patients showed a complete response, three (10%) patients showed a partial response, and one (3.3%) patient had a progressive disease.

Comparison of Dosimetric Parameters of PTV (Mean) and OARs

Table 3 shows the dosimetric parameters of the mean PTV values. The unpaired T-test was used to calculate the p-value. The p-values for none of the parameters were found to be significant which may be due to the fact that in both arms, patients were treated by IMRT techniques.

**Table 3:** Comparison of dosimetric parameters of PTV (mean) PTV: planning target volume, IMRT: intensity-modulated radiotherapy, do-IMRT: dysphagia-optimized IMRT, st-IMRT: standard IMRT, CI: conformity index, HI: homogeneity index.

	st-IMRT (Mean)	do-IMRT (Mean)	p-value
V95 (%)	98.73	98.04	0.25
D2 (Gy)	71.92	72.06	0.17
D50 (Gy)	70.55	70.41	0.13
D95 (Gy)	68.42	68.55	0.24
D98 (Gy)	67.22	67.54	0.13
D100 (Gy)	47.34	49.72	0.27
Dmax (Gy)	73.76	74.02	0.10
Dmean (Gy)	70.36	70.30	0.30
CI	1.20	1.22	0.30
HI	0.06	0.05	0.27

Table 4 shows the comparison of dosimetric parameters of the OARs. The p-values were calculated by the unpaired T-test. The p-value of optic chiasma, right and left optic nerves, right cochlea, and right and left eyes was significant, although, in both arms, the dose constraints of the structures were well within tolerable limits.

**Table 4:** Comparison of dosimetric parameters of OARs OAR: organ at risk, IMRT: intensity-modulated radiotherapy, do-IMRT: dysphagia-optimized IMRT, st-IMRT: standard IMRT, PRV: planning risk volume.

Structure		st-IMRT (Mean)	do-IMRT (Mean)	p-value
Brainstem (Gy)	Dmax	39.31	37.69	0.29
Spinal cord (Gy)	Dmax	39.28	39.53	0.38
PRV spine (Gy)	Dmax	46.21	46.75	0.31
Optic chiasma (Gy)	Dmax	3.29	2.45	0.009
Right optic nerve (Gy)	Dmax	3.30	2.52	0.03
Left optic nerve (Gy)	Dmax	3.02	2.48	0.02
Right parotid gland (Gy)	Dmean	38.29	37.10	0.31
Left parotid gland (Gy)	Dmean	34.51	37.73	0.12
Both parotids (Gy)	Dmean	36.80	36.41	0.43
Right cochlea (Gy)	Dmean	20.95	15.19	0.02
Left cochlea (Gy)	Dmean	16.07	13.46	0.16
Right eye (Gy)	Dmax	3.55	2.73	0.05
Left eye (Gy)	Dmax	3.38	2.97	0.04
Right lens (Gy)	Dmax	2.03	1.75	0.07
Left lens (Gy)	Dmax	1.95	1.74	0.12
Lips (Gy)	Dmean	23.87	22.54	0.31
Mandible (Gy)	Dmax	70.64	71.13	0.39
	Point dose (1cc>75)	68.88	69.23	0.43

Comparison of Volume and Mean to the Structures of DARS

Table 5 shows the comparison of volumes and the mean to the DARS, out of which the p-values of V60 and V70 to the ICM, V60 and V70 to the larynx, V70 and D80 to the UES, and V60 to the DARS were found to be statistically significant.

**Table 5:** Comparison of volume and mean to the structures of DARS CM: constrictor muscles, SCM: superior constrictor muscles, MCM: middle constrictor muscles, ICM: inferior constrictor muscles, BOT: base of tongue, UES: upper esophageal sphincter, DARS: dysphagia aspiration-related structures, IMRT: intensity-modulated radiotherapy, do-IMRT: dysphagia-optimized IMRT, st-IMRT: standard IMRT.

Volumes/Structures		SCM		MCM		ICM		CM		BOT		Larynx		UES		DARS	
		Mean	p	Mean	p	Mean	p	Mean	p	Mean	p	Mean	p	Mean	p	Mean	p
V30	st-IMRT	99.66	0.02	98.55	0.2	99.98	0.16	99.8	0.02	99.98	0.05	99.93	0.11	99.65	0.08	99.89	0.06
	do-IMRT	97.92		99.78		98.01		98.42		98.87		97.55		96.93		94.96	
V50	st-IMRT	92.61	0.49	92.14	0.31	68.72	0.13	84.85	0.12	92.73	0.35	78.32	0.16	64.78	0.16	81.51	0.12
	do-IMRT	92.7		94.6		77.55		88.95		94.44		84.04		55		86.4	
V60	st-IMRT	84.52	0.37	85.65	0.18	44.99	0.01	71.45	0.01	84.84	0.19	59.99	0.009	33.59	0.22	65.37	0.004
	do-IMRT	86.56		91.83		65.44		81.75		90.69		77.35		26.62		79.35	
V70	st-IMRT	63.28	1.05	65.55	6.59	25.66	0.008	51.22	3.41	62.96	6.63	34.24	0.0001	9.69	0.01	40.14	4.44
	do-IMRT	18.5		23.39		7.44		15.89		17.21		10.38		0.86		12.21	
D50	st-IMRT	67.18	0.45	67.05	0.41	56.54	0.1	66.98	0.06	67.04	0.34	62.37	0.31	54.35	0.15	65.55	0.06
	do-IMRT	67.37		67.47		60.25		68.96		67.76		63.79		51.75		67.98	
D80	st-IMRT	63.19	0.39	64.99	0.35	53.28	0.13	56.37	0.22	65	0.39	53.45	0.12	51.56	0.03	54.27	0.13
	do-IMRT	62.24		65.9		56.83		58.46		64.24		57.24		46.65		57.44	
Dmean	st-IMRT	66.91	0.25	67.2	0.42	58.48	0.17	63.77	0.2	67.09	0.42	61.15	0.3	55.05	0.11	62.39	0.14
	do-IMRT	65.76		67.51		60.87		64.78		66.78		63.22		52.15		63.91	

Site-Wise Comparison of Mean Doses to DARS

a) Oropharynx: Table 6 shows the mean doses of DARS and the p-values for oropharyngeal carcinoma cases. None of the p-values were not found to be statistically significant which might be due to the close proximity of the DARS to the PTV.

**Table 6:** Comparison of mean doses of DARS in both groups in the oropharynx CM: constrictor muscles, SCM: superior constrictor muscles, MCM: middle constrictor muscles, ICM: inferior constrictor muscles, BOT: base of tongue, UES: upper esophageal sphincter, DARS: dysphagia aspiration-related structures, IMRT: intensity-modulated radiotherapy, do-IMRT: dysphagia-optimized IMRT, st-IMRT: standard IMRT.

Structures	Group	Mean (Gy)	p-value
SCM	st-IMRT	69.68	0.39
	do-IMRT	69.36	
MCM	st-IMRT	68.64	0.33
	do-IMRT	67.59	
ICM	st-IMRT	57.40	0.42
	do-IMRT	58.07	
CM	st-IMRT	64.68	0.28
	do-IMRT	65.64	
BOT	st-IMRT	69.31	0.43
	do-IMRT	69.04	
Larynx	st-IMRT	60.45	0.36
	do-IMRT	61.36	
UES	st-IMRT	52.43	0.31
	do-IMRT	51.03	
DARS	st-IMRT	62.27	0.28
	do-IMRT	63.45	

b) Oral cavity: Table 7 shows the mean doses of DARS and the p-values for oral cavity carcinoma cases. None of the p-values were found to be statistically significant.

**Table 7:** Comparison of mean doses of DARS in both groups in the oral cavity CM: constrictor muscles, SCM: superior constrictor muscles, MCM: middle constrictor muscles, ICM: inferior constrictor muscles, BOT: base of tongue, UES: upper esophageal sphincter, DARS: dysphagia aspiration-related structures, IMRT: intensity-modulated radiotherapy, do-IMRT: dysphagia-optimized IMRT, st-IMRT: standard IMRT.

Structures	Group	Mean (Gy)	p-value
SCM	st-IMRT	65.48	0.08
	do-IMRT	68.74	
MCM	st-IMRT	61.66	0.31
	do-IMRT	63.68	
ICM	st-IMRT	53.21	0.21
	do-IMRT	49.94	
CM	st-IMRT	60.94	0.21
	do-IMRT	62.68	
BOT	st-IMRT	66.31	0.06
	do-IMRT	69.86	
Larynx	st-IMRT	56.98	0.29
	do-IMRT	54.61	
UES	st-IMRT	53.49	0.42
	do-IMRT	52.66	
DARS	st-IMRT	59.2	0.4
	do-IMRT	59.86	

c) Larynx: Table 8 shows the mean doses of DARS and the p-values for laryngeal carcinoma cases. The p-values of the MCM, ICM, and UES were found to be statistically significant.

**Table 8:** Comparison of mean doses of DARS in both groups in the larynx CM: constrictor muscles, SCM: superior constrictor muscles, MCM: middle constrictor muscles, ICM: inferior constrictor muscles, BOT: base of tongue, UES: upper esophageal sphincter, DARS: dysphagia aspiration-related structures, IMRT: intensity-modulated radiotherapy, do-IMRT: dysphagia-optimized IMRT, st-IMRT: standard IMRT.

Structures	Group	Mean (Gy)	p-value
SCM	st-IMRT	53.25	0.16
	do-IMRT	59.24	
MCM	st-IMRT	71.32	0.005
	do-IMRT	69.54	
ICM	st-IMRT	70.55	0.004
	do-IMRT	68.96	
CM	st-IMRT	61.91	0.25
	do-IMRT	64.28	
BOT	st-IMRT	54.77	0.13
	do-IMRT	63.8	
Larynx	st-IMRT	70.05	0.14
	do-IMRT	69.29	
UES	st-IMRT	65.77	0.01
	do-IMRT	52.47	
DARS	st-IMRT	66.32	0.47
	do-IMRT	66.49	

d) Hypopharynx: Table 9 shows the mean doses of DARS and the p-values for hypopharyngeal carcinoma cases. The p-values of the MCM, ICM, CM, larynx, UES, and combined DARS were all found to be statistically significant.

**Table 9:** Comparison of mean doses of DARS in both groups in the hypopharynx CM: constrictor muscles, SCM: superior constrictor muscles, MCM: middle constrictor muscles, ICM: inferior constrictor muscles, BOT: base of tongue, UES: upper esophageal sphincter, DARS: dysphagia aspiration-related structures, IMRT: intensity-modulated radiotherapy, do-IMRT: dysphagia-optimized IMRT, st-IMRT: standard IMRT.

Structures	Group	Mean (Gy)	p-value
SCM	st-IMRT	69.54	0.09
	do-IMRT	66.23	
MCM	st-IMRT	70.91	0.002
	do-IMRT	69.45	
ICM	st-IMRT	70.63	0.008
	do-IMRT	68.81	
CM	st-IMRT	70.19	0.02
	do-IMRT	67.42	
BOT	st-IMRT	69.85	0.08
	do-IMRT	62.46	
Larynx	st-IMRT	70.45	0.006
	do-IMRT	69.28	
UES	st-IMRT	67.45	0.02
	do-IMRT	53.37	
DARS	st-IMRT	70.23	0.03
	do-IMRT	66.31	

Acute Toxicities of Radiation (RTOG)

In the st-IMRT arm, seven (23.3%) patients had grade 3 mucositis, while in the do-IMRT arm, two (6.6%) patients had grade 3 mucositis. No grade 4 toxicity was seen in either group. In the st-IMRT arm, one (3.3%) patient had grade 3 skin reactions, and in the do-IMRT arm, six (20%) patients had grade 3 skin reactions. No grade 4 toxicity was seen in either group.

Chronic Toxicity of Radiotherapy

At the end of six months post-treatment, none of the patients in the st-IMRT arm had xerostomia, while five (16.6%) patients in the do-IMRT arm had grade 1 xerostomia. At the end of six months post-treatment, nine (30%) patients in the st-IMRT arm had grade 1 or grade 2 dysphagia, while eight (26.6%) patients in the do-IMRT arm had grade 1 dysphagia.

## Discussion

With the advancements of newer radiotherapy techniques of intensity-modulated radiotherapy (IMRT) and image-guided radiotherapy (IGRT), the toxicities associated with radiation are comparatively lesser when compared with the scenario of two decades ago. Due to these advancements, although better conformity is achieved, significant volumes of normal tissues still receive a clinically significant radiation dose.

Radiation results in a significant dose delivery to structures involved in the process of deglutition which subsequently results in dysphagia. A previous study conducted in our department by Upadhyay et al. [5] inferred that IMRT can help in setting goals for optimization and can help in reducing the morbidity of dysphagia better as compared to the 3D-CRT technique. In many recent trials, it has been implied that limiting the dose to the DARS might help in decreasing the incidence and severity of dysphagia. In the present study, we are hereby trying to determine the feasibility to achieve the dose constraints for DARS (without compromising the coverage to the target volumes) and to establish its clinical relevance by using the IMRT technique in the treatment of head and neck cancer patients.

DARS dosimetric parameters

Feng et al. [9] evaluated head and neck cancer patients treated by radiotherapy by intensity-modulated radiotherapy and inferred that there was a statistically significant relationship between dose-volume effect and dysphagia and aspiration. This relationship was used to support the hypothesis that a reduction in dose to the swallowing structures may also help in reducing the prevalence and grade of dysphagia. In our study, the delineation of structures was done as per the guidelines by Christianen et al. [8], and the IMRT technique was used to plan all the cases. The dose constraints given to the DARS were the same as defined in the study by Joseph et al. [10]. In the combined constrictor muscles, the p-values of the V30 and V60 were significant. A relative reduction in the doses was also seen in the dysphagia-optimized arm as compared to the arm in which no optimization was given to the DARS.

The present study is the continuation of the thought process of evaluating doses to the DARS done in the department and published by Upadhyay et al. [5] which evaluated the Dmean, V30, V50, V60, D50, and D80 doses to the pharyngeal constrictor muscles and the combined DARS. The results showed that the doses received by the IMRT arm were significantly lower as compared to the 3D-CRT arm, hence showing that there was lesser worsening of dysphagia in the IMRT arm. The present study therefore was done only in IMRT patients to try to define the doses of the DARS. The other parameters included were V70 and Dmean. In our study, even though the mean dose to the DARS was more than 63 Gy, no grade 3 or 4 dysphagia was seen which may be due to the fact that dysphagia could be most commonly affected by the site of involvement and the swallowing structure lying closest to the PTV.

Tyler et al. [11] concluded that using larger CTV-PTV margins was associated with high doses of the DARS due to a greater PTV overlap and their constraints not being mandatory. The use of smaller PTV margins (3 mm) as compared to the 5 mm margin (if the institutional protocol allows) can help in sparing the DARS. Calcuttawala et al. [12] inferenced in their study that the larger PTVs lead to the increased overlap volumes of PTV and DARS; hence, achieving constraints below 50 Gy becomes difficult. Larger PTVs may be a reason for not being able to achieve the DARS constraints in our study. Another study done in our department by Kumar et al. [13] shows the role of decreasing PTV margins to decrease dose to the OARs. The study reveals that decreasing the PTV margins to 4 mm can help to reduce the dose of the parotids significantly, but the implementation of the same needs to be supplemented by daily imaging. The same concept can be used here, by reducing the PTV margin to 3 mm instead of using a 5 mm margin which may help in the dose reduction to the DARS by reducing the overlap between the PTV and DARS. The reduction of PTVs should be undertaken as an institutional short study to see the feasibility. Further, the incorporation of better immobilization devices and image guidance may significantly help in achieving the dose constraints of the DARS.

Charters et al. [14] in their meta-analysis concluded that a mean dose of more than 50 Gy to the constrictor muscles and larynx was associated with dysphagia. They also stated that proton therapy may be better equipped to provide better conformity. Calcuttawala et al. [12] stated in their study that DARS optimization could be a good treatment strategy in patients with a smaller volume of high-risk PTV. In N2c disease status, bilateral neck irradiation and PTV lying in close proximity to the DARS make it difficult to spare the DARS. In our study, due to simple randomization, we did not randomize patients based on the site or tumor stage, and the mean dose of 64.78 Gy to the constrictor muscles and a mean dose of 63.91 Gy to the DARS were observed in the dysphagia-optimized arm, while the standard arm received 63.77 Gy to the constrictor muscles and 62.39 Gy to the DARS. A larger dose in some of the cases can be seen if we even compare two of the same sites in both arms; this fact could be attributed to a larger PTV in terms of larger tumor size, nodal burden of disease, and bilateral neck irradiation. We suggest that achieving a dose less than 50 Gy is not feasible, until there is a less geographical proximity between the PTV and DARS and lesser dose prescription of around 54-60 Gy. In clinical scenarios of post-operative cases or low-risk volumes of neck node for irradiation, there is possibility of minimizing the doses to DARS and thereby achieving the doses within the defined constraints. We suggest that in node-negative disease or by de-escalation of dose to the low- or intermediate-risk areas especially in human papilloma virus (HPV)-positive oropharyngeal cancer patients, positive upper cervical nodes can help de-escalate the dose to the lower neck level which may help in reducing the dose to the individual DARS and thus can help in driving down the overall mean dose.

Teguh et al. [15] in their study inferenced that using brachytherapy and Cyberknife to provide a boost after a dose of 46 Gy can lead to an improvement in dysphagia-related problems. Cyberknife also helped to provide a higher dose to the CTV, and also the PTV margins have a significant effect on the dose of the swallowing muscles. Caudell et al. [16] in their study found that Dmean more than 41 Gy, V60 more than 24% to the larynx, V60 more than 12% to the ICM, and V65 more than 33% to the SCM or more than 75% to the MCM result in the development of pharyngoesophageal stricture requiring dilation and increase the risk of aspiration. Li et al. [17] in their study defined dosimetric parameters for DARS and found a correlation between the dose reduction to the ICM and cricopharyngeal inlet and the need for gastrostomy tube dependence. In our study, there was a reduction in the number of patients who required Ryle’s tube placement post-treatment in the do-IMRT arm as there was a relative reduction in the amount of dose received by the DARS in the do-IMRT arm, although the required dose constraints could not be met in the majority of the cases. As per the study by Teguh et al. [15] to meet the required dose constraints, IMRT could be combined with brachytherapy or Cyberknife techniques to help in reducing the dose to the DARS as these techniques have a rapid fall-off dose gradient and smaller PTV margins; hence, a reduction in the dose can be seen in the DARS leading to a better swallowing outcome for the patient.

In a study by Grover et al. [18], they stated that patients with locally advanced disease had higher chances of developing dysphagia with secretion pooling being the most common dysfunction. They stated that fiberoptic endoscopic evaluation (FEES) evaluation can help in reducing mortality due to silent aspiration. Lewin [19] recommended that starting swallowing therapy at treatment initiation and continuing the therapy throughout the course of the treatment may help prevention of resulting in long-term dysphagia. In our study cohort, no swallowing therapies were advised to the patients and evaluation by FEES was also not performed. However, as shown by various studies, early rehabilitation in the form of swallowing exercises and evaluation by FEES can help in the reduction of mortality in the patients as timely intervention can be performed.

Clinical toxicities post-radiation

Nutting et al. [20] stated that by using parotid-sparing IMRT techniques, a reduction in radiation-induced late toxicities can be achieved for xerostomia and also can help in the reduction of dysphagia. Joseph [21] in his study showed that a reduced duration of grade 2 and grade 3 dysphagia is seen in patients treated with IMRT-simultaneous integrated boost (SIB) technique which can lead to a faster recovery of nutritional status. In our study population, 91.6% of the patients in the entire cohort showed no xerostomia, while 16.6% showed grade 1 xerostomia at the end of six months. In terms of dysphagia, no toxicity was seen in 73.3% of the patients in the dysphagia-optimized arm, while 26.6% of the patients had grade 1 toxicity. No grade 2 above dysphagia was seen in the dysphagia-optimized arm. In the standard arm, 16.6% of the patients had grade 1 toxicity and 13.3% of the patients had grade 2 toxicity. By using IMRT techniques, the contralateral parotid gland can be spared which helps in reduced xerostomia which can help to reduce dysphagia as adequate production helps in proper lubrication of food which allows for easier deglutition. In terms of dysphagia, optimizing the dose to the DARS may be the reason for lesser reactions in the dysphagia-optimized arm. By optimizing the dose to the DARS, the severity of dysphagia is lesser which helps in a relatively faster recovery so that the nutritional status can be adequately maintained for the patient.

Ryle’s tube dependence

Nguyen et al. [22] performed modified barium swallow (MBS) studies to assess the prevalence of aspiration before and after chemoradiation. They concluded in their study that performing diagnostic studies was necessary to overcome the morbidity associated with silent aspiration. To help overcome dysphagia, Rosenthal et al. [23] made recommendations about therapeutic interventions that can be undertaken which showed improvement in their patients undergoing radiotherapy. If left unaddressed, Denaro et al. [24] stated that approximately one-third of the patients with dysphagia developed pneumonia which was in turn associated with a high mortality rate. As dysphagia is related to great physical and mental anguish to the patient, Shen et al. [25] stated that manufacturing texture-improved foods could help in reducing the incidence of malnutrition in patients due to dysphagia. In our study population in st-IMRT, 23.3% of the patients underwent Ryle’s tube placement pre-treatment and 33.3% of the patients in this group required Ryle’s tube post-treatment for a period of six weeks, while in do-IMRT, 10% of the patients underwent Ryle’s tube placement during treatment, while none of the patients required Ryle’s tube post-treatment completion. In our study, the limitation was that we did not perform any assessment of the degree of dysphagia which could contribute toward aspiration, and also no therapeutic interventions were recommended to the patients, but dietary consultation was done in terms of improving the texture of foods, which can help in not only reducing the incidence of malnutrition in patients but also aids in reducing the mental anguish in patients that occurs due to the dependence on tube feeding.

Site-wise comparison of DARS dosimetry

Langendijk et al. [26] stated that the close proximity of the retropharyngeal and parapharyngeal spaces to the CTV in oropharyngeal and nasopharyngeal cancers contributes to a relatively higher dose to the constrictor muscles. Frowen et al. [27] stated that patients with larger tumor sizes, bilateral neck irradiation, and hypopharyngeal cancers had worse outcomes in terms of dysphagia when compared to oropharyngeal and laryngeal cancers. Shune et al. [28] in their study predicted that hypopharyngeal and oral cavity tumors and dysphagia were strong predictors of prognosis. Maintenance of oral hygiene might negatively impact dysphagia. In our study, we only used the IMRT technique for treatment delivery of the entire dose. In cases of oropharyngeal and hypopharyngeal cancer patients, a high dose was being received by the constrictor muscles. Patients were followed on a weekly basis and regularly counseled on the need to maintain oral hygiene by the usage of chlorhexidine gargles and antifungal medications.

Van der Molen et al. [29] in their study stated that the mean doses of one structure had a strong correlation which is in close proximity/adjacent to it rather than a structure which is relatively further. Van der Laan et al. [30] stated that dosimetry to the DARS could be improved by increasing efforts toward the reduction in the DARS and also by accepting the plans which shift the dose to other unspecified normal tissues so as to spare the DARS. They also inferenced that do-IMRT was significantly dependent on the location of the primary tumor site, neck irradiation, and the amount of overlap between the PTV and DARS. In all the cases in our study, all patients underwent bilateral neck irradiation and dysphagia-optimized arm had a greater number of hypopharyngeal and laryngeal cancer patients. Efforts should be made to critically analyze the plans so as to spare the DARS even if an acceptable PTV coverage has been achieved. Replanning may be required so as to allow for dose dumping/shifting the dose to other surrounding structures which may help in the reduction of dose received by the swallowing structures. A similar concept has already been published by Khattar et al. [1] in terms of reducing mucositis during head and neck irradiation by delineation of pseudo-OAR and decreasing the dose dumping to the undefined normal tissues.

Petkar et al. [31] in their trial emphasized an important point that the degree by which any individual subset of structure is spared will depend on the subsite involved and also the importance of each structure which needs to be optimized will also change depending on the primary tumor location due to their geographical proximity. They stated that swallowing improvements may be achievable through technological advancements, but the eradication of the issue completely is unlikely. In our study, a higher dose to the constrictor muscles and the larynx could be accounted for due to its close proximity to the high-risk CTVs. Although a relative reduction of the doses was seen when these structures were optimized. A greater number of patients in the hypopharynx and larynx arm could possibly account for a relatively higher overall dose in the dysphagia-optimized arm. If the dose to one of the structures was higher, it had an impact on the mean dose of the adjacent structure; hence, this geographical proximity could also be the reason for higher doses to the constrictor muscles and DARS depending on the site involved and the structure receiving a high dose driving up the overall mean dose. The author suggests that besides focusing on the mean doses to the DARS, dose optimization/reduction should be considered for individualized units of DARS (SCM, MCM, ICM, BOT, larynx, and UES). If any subunits of DARS are geographically far away from the PTV or are lying in a low-risk volume of CTV neck, it may help to decrease the dose to individual DARS and hence the overall mean dose to the DARS.

## Conclusions

Dose constraints to DARS cannot be achieved in all head and neck cancer patients. It is limited by the stage of the tumor which may have higher PTVs and also due to the site of the tumor causing close proximity to the DARS. Further dose prescription may also be a reason to not achieve the dose constraints to DARS. Therefore, patient selection is important where it can be tried in post-operative patients (where the dose prescription is less than the de-novo patients) and low-risk neck node CTVs (where the dose prescription is lesser than high-risk nodal regions). Further, where PTV margins are at a distance from DARS, there are better chances to achieve dose constraints to DARS with slightly lesser prescriptions to PTV. Last but not least, patients should be encouraged to maintain oral hygiene, and swallowing exercises and early enteral nutrition rehabilitation should also be done to prevent atrophy of the swallowing muscles.
